# Modifiable lifestyle and metabolic risk factors for colorectal polyps: a systematic review and meta-analysis

**DOI:** 10.3389/fpubh.2025.1655750

**Published:** 2025-10-08

**Authors:** Yue Lei, Zihong Cai, Mengli Zheng, Yunfan Deng, Shirui Li, Xiuyang Li, Sheng Dai

**Affiliations:** ^**1**^Department of Big Data in Health Science, and Center for Clinical Big Data and Statistics, the Second Affiliated Hospital, College of Medicine, Zhejiang University, Hangzhou, China; ^**2**^Department of General Surgery, Affiliated Sir Run Run Shaw Hospital, Zhejiang University School of Medicine, Hangzhou, China

**Keywords:** colorectal polyps, pathological types, meta-analysis, risk factors, lifestyle

## Abstract

**Objective:**

To evaluate associations between unhealthy lifestyles, metabolic diseases, and colorectal polyps, with emphasis on subtype-specific effects.

**Methods:**

We systematically searched PubMed, Embase, Cochrane Library, and SinoMed (up to July 2024) for studies reporting odds ratios (ORs) of colorectal polyps associated with lifestyle or metabolic factors. Heterogeneity was quantified using I (2) statistics, with random-effects models applied as the primary analytical approach. Subgroup analyses were conducted to investigate potential effect modifiers, and meta-regression was performed to explore continuous sources of heterogeneity, while sensitivity analyses and funnel plots evaluated robustness and bias.

**Results:**

Alcohol (OR = 1.63, 95%CI:1.48-1.78), high-fat diet (OR = 1.45, 95%CI:1.33-1.57), and smoking (OR = 1.79, 95%CI:1.69-1.90) significantly increased polyp risk across subtypes. Smoking showed subtype- and region-specific effects, with the highest risk for sessile serrated lesions (SSLs; (OR = 3.06, 95%CI:2.41-3.90)) and in the US, South Korea, and Israel. Type 2 diabetes had the strongest metabolic association (OR = 2.17,95%CI:1.82- 2.60), followed by hyperlipidemia (OR = 1.50, 95%CI:1.32-1.70) and hypertension (OR = 1.33, 95%CI:1.10-1.61). Heterogeneity stemmed from pathological classification and geographic variation, with no significant publication bias.

**Conclusion:**

Unhealthy lifestyles (alcohol, high-fat diet, smoking) and metabolic diseases (type 2 diabetes, hyperlipidemia, hypertension) independently increase colorectal polyp risk, with smoking demonstrating pronounced subtype and regional variability. These findings can inform the development of risk-stratified screening protocols and targeted public health interventions.

## Highlights

- First meta-analysis quantifying smoking’s subtype-specific effects on SSLs (OR = 3.06).
- Alcohol, high-fat diet, and smoking all independently elevate colorectal polyp risk.
- Type 2 diabetes shows strongest metabolic association (OR = 2.17) among comorbidities.
- Reveals geographic heterogeneity in risk factors (US/S. Korea/Israel most affected).

## 1 Introduction

Colorectal cancer (CRC) accounts for over 1.9 million new cases annually worldwide, with 80% arising from precursor polyps (1, 2). Most sporadic cases of CRC progress through the adenoma-carcinoma sequence, advancing from dysplastic epithelium to malignancy, while recent evidence suggests sessile serrated lesions (SSLs) may represent a distinct malignant pathway (3, 4). This heterogeneity underscores the need to elucidate differential risk factors across polyp subtypes – a gap persisting in current literature.

Although unhealthy lifestyles and metabolic diseases are implicated in CRC pathogenesis, their subtype-specific effects remain controversial. For instance, while smoking consistently associates with adenomas, its impact on SSLs varies significantly across cohorts (5, 6). Similarly, type 2 diabetes demonstrates stronger associations with advanced adenomas than hyperplastic polyps (HPs), but limited studies have comprehensively analyzed the associations between unhealthy lifestyles and metabolic diseases and distinct pathological subtypes of colorectal polyps (7, 8). To address these inconsistencies, we performed the first comprehensive meta-analysis stratifying by polyp subtypes (adenomas, SSLs, HPs) and geographic regions. Specifically, we quantified differential effects of modifiable risk factors (smoking, alcohol, and diet); assessed metabolic diseases’ subtype-specific risks; and explored regional variations in these associations, thereby providing clinically relevant evidence to inform targeted prevention and region-specific screening strategies.

## 2 Materials and methods

### 2.1 Search strategy

**Figure 1:**
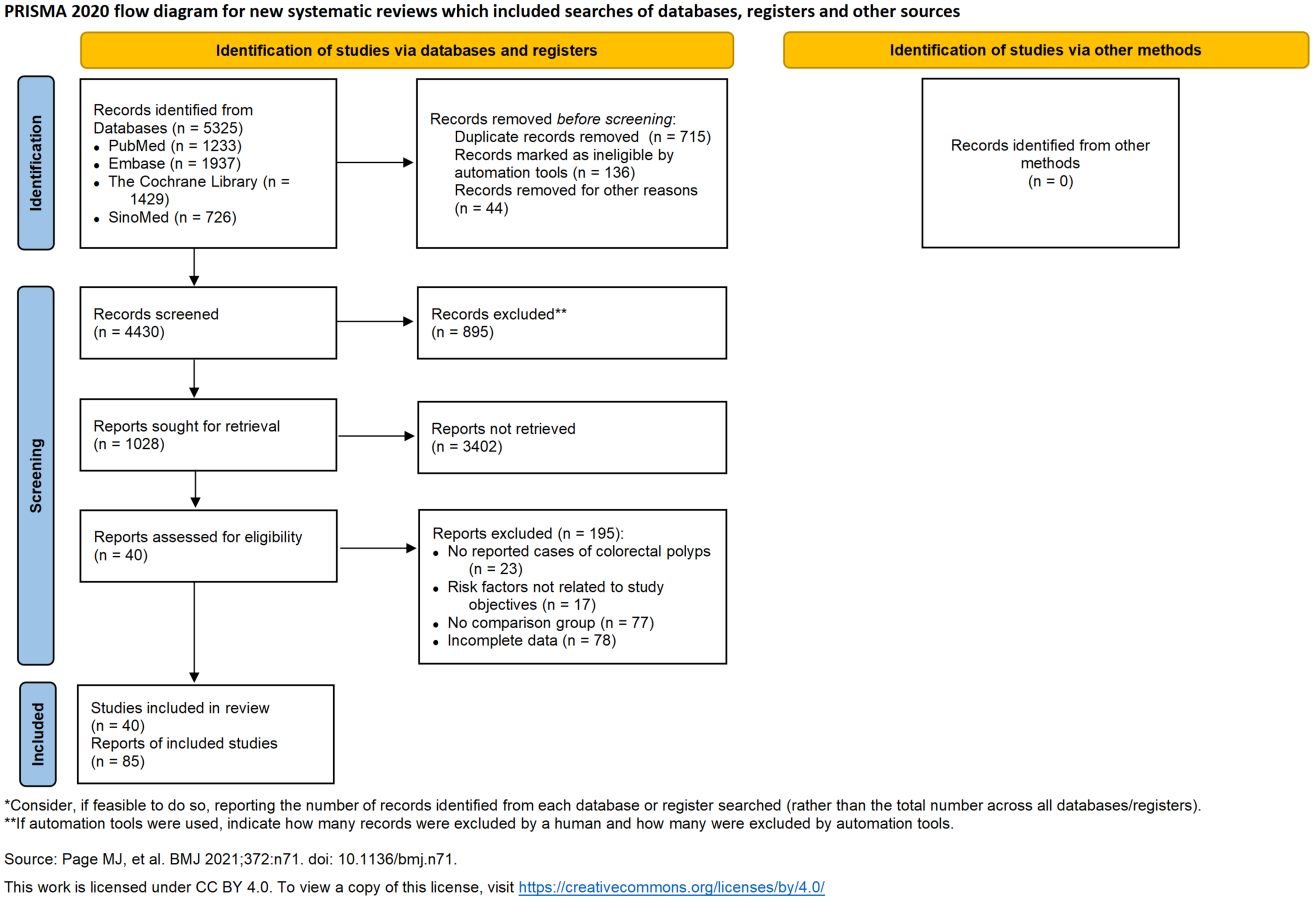
PRISMA flow diagram for the identification and selection of studies.

This systematic review and meta-analysis was conducted following the guidelines set forth by the Cochrane Handbook for Systematic Reviews of Interventions and the Preferred Reporting Items for Systematic Reviews and Meta-Analyses (PRISMA) guidelines (9). We searched the PubMed, Embase, Cochrane Library, and Chinese Biomedical Literature Database (SinoMed). These databases were chosen as they represent the most comprehensive and authoritative repositories of international and Chinese biomedical literature, ensuring a broad and inclusive search. Our approach to gray literature involved including relevant conference abstracts found within these databases to capture emerging research. However, to ensure a high standard of methodological quality for our analysis, other forms of unpublished data were not included. Additionally, we reviewed the International Clinical Trials Registry Platform^1^ to identify ongoing trials and minimize the risk of overlapping studies. Our search targeted studies on risk factors associated with different pathological types of intestinal polyps, with a search range extending from database inception to July 25, 2024 (Figure 1).

The search terms included at least one of the following keywords or subject terms. In Chinese, these included “serrated polyps,” “hyperplastic colorectal polyps,” “inflammatory polyps,” “polyp,” “adenoma,” “colorectal,” “pathological type,” “risk factor,” and “influencing factor.” English keywords included “risk factor,” “serrated,” “hyperplastic,” “polyp,” “adenoma,” “colorectal,” “rectum,” and their synonyms and derivatives. Titles and abstracts of the retrieved studies were cross-checked independently by three researchers to ensure thorough and accurate identification of relevant studies.

### 2.2 Inclusion and exclusion criteria

Following the PICOS (Population, Intervention, Comparison, Outcome, and Study Design) criteria, studies meeting the following inclusion criteria were selected: (1) studies examining the impact of unhealthy lifestyle habits on colorectal polyps; (2) studies exploring the association between metabolic diseases and colorectal polyps; (3) studies addressing factors influencing different pathological types of colorectal polyps; (4) studies involving participants aged ≥18 years, without gender restrictions; (5) studies providing sufficient data to calculate effect sizes (e.g., odds ratio, relative risk, or hazard ratio) and corresponding 95% confidence intervals.

Exclusion criteria were as follows: (1) studies involving populations with other intestinal diseases or comorbidities, such as Crohn’s disease, ulcerative colitis, or colorectal cancer; (2) case reports, letters, reviews, and animal or cell experiments; (3) duplicate publications; (4) studies not published in Chinese or English; and (5) studies lacking specific effect values or original data, precluding meta-analysis.

### 2.3 Data collection and quality assessment

Three researchers (Y. L., ZH. C., and ML. Z.) independently screened the literature using NoteExpress or Zotero software. Full-text articles and abstracts were reviewed to identify all relevant studies to be included, and a standardized data extraction form was created to organize study details. Detailed information from all included studies was recorded, including first author, publication year, study region, sample size, polyp pathology type, population characteristics, study groupings, influencing factors, and OR values. Any discrepancies were initially discussed among the three researchers to reach a consensus. If a consensus could not be reached, a fourth researcher (XY. L.) was consulted to make a final decision. The final studies were collected using Review Manager 5.4, and the Newcastle-Ottawa Scale was used to assess study quality (10).

### 2.4 Statistical analysis

The present study employed a staged approach to examine risk factor associations with colorectal polyps. First, we performed separate meta-regression for lifestyle factors (alcohol, smoking, high-fat diet) and metabolic diseases (T2DM, hyperlipidemia, hypertension), calculating pooled odds ratios (ORs) with 95% confidence intervals. Heterogeneity was quantified via I (2) statistics, with I^2^ > 50% indicating substantial heterogeneity warranting random-effects modeling (11). Second, we performed subgroup analyses specifically targeting significant covariates identified in meta-regression (*P* < 0.05), stratified by pathological type (adenomas, SSLs, HPs) and geographic region. Forest plots were generated to visualize individual study estimates and pooled effect sizes for each subgroup (12).

Methodological rigor was ensured through two validation steps: (1) Funnel plots with Egger’s test (threshold *P* < 0.10) assessed publication bias; (2) Leave-one-out sensitivity analyses evaluated effect size stability. All statistical analyses were performed using Review Manager 5.4 and STATA 17.0 software.

## 3 Results

### 3.1 Literature screening and study characteristics

The PRISMA-compliant selection process (Figure 1) identified 5,325 records, with 41 studies meeting inclusion criteria. Included studies from 8 countries, predominantly China, the US, and South Korea. The basic characteristics of the 41 included studies are presented in Table 1. Newcastle-Ottawa Scale (NOS) scores ranged 5–8 (Table 2), indicating high quality.

**Table 1:** Basic characteristics of included studies.

Study	Study period	Region	Case count (case/population)	Age/year	Pathological types	Associated factors	OR (95%CI)
Bai 2024 (30)	2020–2022	China	166/550	Mean: 44.08(Control) and 56.36(Case)	*Other Types*	*Current Smoking*	1.47 (1.18–1.83)
Chen 2017 (31)	2014–2016	China	934/2409	Mean: 44.65(Control) and 50.20(Case)	*Other Types*	*Current Alcohol Consumption*	1.28 (1.04–1.57)
					*Other Types*	*Current Smoking*	1.83 (1.53–2.19)
Chi 2021 (32)	2013–2019	China	668(Adenoma); 385(SSLs)	Mean: 53	*Adenoma*	*Current Alcohol Consumption*	1.84 (1.08–3.15)
					*Other Types*	*Current Alcohol Consumption*	1.84 (0.97–3.51)
					*Other Types*	*Current Smoking*	1.52 (1.09–2.12)
Davenport 2016 (33)	2003–2010	the U. S.	1779(Adenoma); 560(HP); 214(SSLs); 3,851(Control)	Mean: 57.2(Control) 59.0(Adenoma) 56.8(HPs) 57.8(SSLs)	*Adenoma*	*Current Smoking*	2.68 (2.19–3.28)
					*Serrated Polyps*	*Current Smoking*	4.68 (2.99–7.32)
					*Adenoma*	*High-Fat Diet*	1.53 (1.21–1.94)
					*Serrated Polyps*	*High-Fat Diet*	2.59 (1.41–4.75)
Dong 2021 (34)	2017–2018	China	312/2000	Mean: 49.78(control) and 51.34(case)	*Adenoma*	*Current Alcohol Consumption*	1.85 (1.23–2.79)
					*Adenoma*	*Current Smoking*	1.79 (1.19–2.70)
					*Adenoma*	*High-Fat Diet*	1.36 (1.02–1.83)
					*Adenoma*	*Dyslipidemia*	1.72 (1.24–2.40)
Erhardt 2002 (35)	1995–1997	Germany	207(Adenoma); 71(HPs); 224(Control)	Male: Mean 61(Adenoma) and 58(HPs) and 54.5(control) Female: Mean 66(Adenoma) and 59.5(HPs) and 55.5(Control)	*Adenoma*	*Current Smoking*	1.56 (1.01–2.40)
Fu 2011 (36)	2003–2010	the U. S.	622(HPs); 1881(Adenoma); 3,764(Control)	Range: 40–75	*Adenoma*	*High-Fat Diet*	1.40 (1.21–1.62)
					*Hyperplastic Polyps*	*High-Fat Diet*	1.30 (1.00–1.69)
Fu 2012 (37)	2003–2011	the U. S.	2543/3764	Mean: 56.8(Control) 58.6(Adenoma) 56.7(HPs) 58.2(Adenoma + HPs)	*Adenoma*	*High-Fat Diet*	1.36 (1.04–1.78)
					*Hyperplastic Polyps*	*High-Fat Diet*	1.38 (1.13–1.69)
					*Other Types*	*High-Fat Diet*	1.61 (1.15–2.26)
Hu 2019 (38)	2018–2019	China	290/608	Mean 53.46(Control) and 58.14(Case)	*Adenoma*	*Current Smoking*	0.98 (0.62–1.56)
Hassan 2010 (39)	2002–2003	Italy	378(Adenoma); 157(HPs)	57.8 ± 7.3(SD)	*Hyperplastic Polyps*	*Current Smoking*	1.98 (1.41–2.78)
Joo 2009 (40)	2002–2008	Korea	186/372	Mean: 63.0	*Adenoma*	*T2DM*	1.90 (1.06–3.41)
Lee 2019 (41)	2012–2014	Korea	12,128(Adenoma); 946(SSPs)	Range: 30–75	*Serrated Polyps*	*Current Alcohol Consumption*	1.86 (0.83–4.17)
					*Serrated Polyps*	*Current Smoking*	3.98 (2.38–6.65)
Leonardo Zorron 2020 (7)	2016–2018	Australia	291(all): 168(Adenoma); 45(SSPs)	Mean: 63.9	*Adenoma*	*Current Smoking*	2.24 (1.17–4.28)
					*Serrated Polyps*	*T2DM*	3.12 (1.53–6.35)
Li 2018 (42)	2016	China	239/484	Mean: 54.6(Control) and 59.7(Case)	*Other Types*	*Dyslipidemia*	1.63 (1.02–2.60)
Li 2020 (43)	2017–2019	China	1439/1717	Mean: 50.65(Control) 58.55(Adenoma) 56.88(Non-adenoma)	*Adenoma*	*Current Alcohol Consumption*	1.35 (1.04–1.76)
					*Other Types*	*Current Alcohol Consumption*	2.19 (1.26–3.80)
					*Other Types*	*Current Smoking*	2.45 (1.51–3.97)
Lingling Liu 2019 (44)	2018–2019	China	615(all): 206(polyps); 138(adenoma)	Mean 47.38 Range 18–81	*Adenoma*	*Current Alcohol Consumption*	1.43 (0.89–2.30)
					*Other Types*	*Current Alcohol Consumption*	1.35 (0.89–2.06)
					*Adenoma*	*Current Smoking*	1.67 (1.11–2.52)
					*Other Types*	*Current Smoking*	1.43 (0.90–2.27)
Liu 2018 (45)	2001–2009	China	909(Adenoma); 651(Others); 5,506(Control)	49.4 ± 12.4 (Control) 51.8 ± 11.4 (Others) 55.5 ± 11.0 (Adenoma)	*Adenoma*	*Current Smoking*	1.35 (1.08–1.71)
					*Other Types*	*Current Smoking*	3.01 (1.91–4.75)
Lu 2020 (46)	2016–2019	China	188/724	Mean: 58.25(control) and 59.62(case)	*Other Types*	*Dyslipidemia*	1.83 (0.99–3.38)
Mosley 2020 (47)	2003–2010	the U. S.	7,621(All): 1787(Adenoma); 59(HPs); 212(SSPs)	57.2 (56.7, 58.8)	*Serrated Polyps*	*High-Fat Diet*	2.38 (1.44–3.93)
Naomi Fliss-Isakov 2017 (48)	2010–2015	Israel	828(All): 348(Adenoma); 73(SSPs); 406(Control)	Range: 40–70	*Adenoma*	*Current Smoking*	3.01 (1.91–4.75)
					*Serrated Polyps*	*Current Smoking*	6.36 (2.77–14.59)
					*Other Types*	*Current Smoking*	3.01 (1.91–4.75)
Omata 2009 (49)	2009	Japan	194(CRN); 132(HP); 42(CRN + HP);586(Control)	Mean: 52.2	*Other Types*	*Current Alcohol Consumption*	1.53 (0.95–2.45)
					*Hyperplastic Polyps*	*Current Smoking*	1.95 (1.21–3.13)
Qin 2021 (50)	2019–2020	China	262(All) 113(Adenoma); 33(ICPs); 116(Control)	Mean: 58.34(control) and 59.34(case)	*Adenoma*	*Current Alcohol Consumption*	1.65 (0.77–3.57)
					*Other Types*	*Current Alcohol Consumption*	1.71 (0.82–3.55)
					*Adenoma*	*Current Smoking*	2.05 (0.89–4.72)
					*Other Types*	*Current Smoking*	1.86 (0.83–4.17)
Rashid *N. lui* 2020 (51)	2008–2014	China	82/3308	Mean: 60(control) and 57(case)	*Serrated Polyps*	*Current Smoking*	2.50 (1.01–6.17)
					*Serrated Polyps*	*T2DM*	2.70 (1.11–6.56)
					*Serrated Polyps*	*T2DM*	2.40 (1.28–4.51)
					*Serrated Polyps*	*Hypertension*	1.30 (0.78–2.16)
Santiago 2021 (52)	2016–2020	the U. S.	816/1370	60.73 ± 8.63 (SD)	*Serrated Polyps*	*Hypertension*	1.39 (0.89–2.18)
Shaohua Zhang 2023 (53)	2021–2022	China	385/792	Mean: 52.21(Control) and 56.60(Case)	*Other Types*	*Current Alcohol Consumption*	1.17 (0.64–2.13)
Shi 2020 (54)	2019	China	168/337	Mean: 53.10(Control) and 53.90(Case)	*Other Types*	*Current Smoking*	1.93 (0.86–4.34)
					*Other Types*	*Dyslipidemia*	2.11 (1.08–4.12)
Suh 2011 (55)	1995–2009	Korea	509/1004	Mean: 55.83(Control) and 55.98(Case)	*Adenoma*	*T2DM*	2.85 (1.83–4.43)
Wang 2014 (56)	2009–2011	China	520/1379	Mean: 52.8	*Adenoma*	*Current Smoking*	1.38 (1.03–1.85)
Wang 2022 (57)	2015–2018	China	532/2996	Mean: 54.97(control) and 56.44(case)	*Other Types*	*Current Alcohol Consumption*	2.39 (1.03–5.55)
Wang 2023 (58)	2021–2022	China	91/151	Mean: 49.93(control) and 52.87(case)	*Other Types*	*Dyslipidemia*	1.37 (0.74–2.55)
Watanabe 2015 (59)	2007–2010	Japan	577/1318	Mean: 59.7	*Other Types*	*Current Alcohol Consumption*	1.35 (1.05–1.74)
					*Other Types*	*Current Smoking*	1.66 (1.27–2.16)
Wu 2021 (60)	2019–2020	China	168/248	Mean: 46.8(control) and 48.3(case)	*Other Types*	*Dyslipidemia*	2.55 (1.12–5.81)
Xing 2021 (61)	2013–2014	China	140/662	Mean: 55.07(control) and 57.43(case)	*Adenoma*	*Current Alcohol Consumption*	1.63 (1.07–2.46)
					*Adenoma*	*Current Smoking*	1.79 (1.09–2.94)
Ye 2018 (62)	2014–2015	China	254/404	Mean: 54.60(control) and 49.51(case)	*Other Types*	*Dyslipidemia*	0.43 (0.08–2.25)
Yu 2021 (63)	2014–2017	China	1379/4514	Mean: 45(control) and 50(case)	*Adenoma*	*Current Smoking*	1.30 (1.06–1.60)
					*Other Types*	*Current Smoking*	1.67 (1.41–1.98)
					*Adenoma*	*Dyslipidemia*	1.34 (1.08–1.67)
					*Other Types*	*Dyslipidemia*	1.31 (1.01–1.70)
Zhan 2016 (64)	2004	Germany	350/839	Mean: 64.8	*Other Types*	*Current Smoking*	2.04 (1.25–3.32)
						*High-Fat Diet*	3.61 (1.00–13.00)
Zhang 2021 (65)	2013–2014	China	166(All) 73(Adenoma); 30(Non-adenoma); 93(Control)	Mean: 55.37(control) 59.26(adenoma) 56.90(non-adenoma)	*Adenoma*	*Current Alcohol Consumption*	2.32 (0.95–5.62)
					*Other Types*	*Current Alcohol Consumption*	1.81 (0.64–5.16)
					*Adenoma*	*Current Smoking*	1.89 (0.64–5.59)
					*Other Types*	*Current Smoking*	4.05 (1.27–12.91)
					*Adenoma*	*Hypertension*	1.65 (0.73–3.73)
					*Other Types*	*Hypertension*	1.76 (0.67–4.58)
Zhang 2023 (66)	2010–2020	China	2,295(All): 459 (SSLs); 918 (Adenomas)	Mean: 65.4	*Serrated Polyps*	*Current Alcohol Consumption*	1.79 (0.89–3.62)
					*Serrated Polyps*	*Current Smoking*	1.53 (1.00–2.33)
					*Serrated Polyps*	*T2DM*	1.82 (1.34–2.48)
					*Serrated Polyps*	*Hypertension*	1.52 (1.07–2.16)
Zhao 2018 (67)	2014–2017	China	358/630	Mean: 61.28(control) and 64.77(case)	*Adenoma*	*Current Smoking*	1.53 (1.08–2.17)
					*Adenoma*	*T2DM*	2.00 (1.09–3.68)
					*Adenoma*	*Hypertension*	1.04 (0.72–1.49)
Zhao 2023 (68)	2021–2023	China	180/451	Mean: 49.3(control) and 51.52(case)	*Other Types*	*T2DM*	2.10 (1.28–3.43)

**Table 2:** Risk of bias assessment for included studies (NOS score).

Included study	Study population selection (0–4)	Comparability (0–2)	Exposure/outcome measurement (0–3)	Total score (0–9)
Bai 2024	4	0	2	6
Chen 2017	4	2	1	7
Chi 2021	4	2	1	7
Davenport 2016	4	2	1	7
Dong 2021	4	0	2	6
Dong 2021	4	0	2	6
Erhardt 2002	4	2	2	8
Fu 2011	4	2	2	8
Fu 2012	4	2	2	8
Hu 2019	4	2	2	8
Joo 2009	4	2	2	8
Lee 2019	3	2	2	7
Leonardo Zorron 2020	4	2	0	6
Li 2018	4	0	2	6
Li 2020	4	0	2	6
Lingling Liu 2019	4	0	2	6
Liu 2018	4	0	2	6
Lu 2020	4	0	2	6
Mosley 2020	4	2	1	7
Naomi Fliss-Isakov 2017	4	2	0	6
Omata 2009	3	2	1	6
Qin 2021	4	2	1	7
Qin 2021	4	2	2	8
Rashid N. Lui 2020	3	0	2	5
Santiago 2021	4	2	2	8
Shaohua Zhang 2023	4	0	1	5
Shi 2020	4	0	2	6
Suh 2011	4	1	1	6
Thakkar 2010	4	0	1	5
Wang 2014	4	2	1	7
Wang 2022	4	0	2	6
Wang 2023	4	0	2	6
Watanabe 2015	4	0	2	6
Wu 2021	4	0	2	6
Xing 2021	4	0	1	5
Ye 2018	4	0	2	6
Yu 2021	4	2	2	8
Zhan 2016	3	1	2	6
Zhang 2021	4	0	2	6
Zhang 2023	4	2	0	6
Zhao 2018	4	0	2	6
Zhao 2023	4	0	2	6

### 3.2 Primary meta-regression results

Table 3 presents the staged analysis results, unhealthy habits as a composite exposure (Coeff = 0.157, *P* = 0.010) that integrated alcohol consumption, smoking, and high-fat diet. For metabolic diseases, disease type emerged as the predominant factor (Coeff = −0.251, *P* = 0.001). Subsequent subgroup analyses revealed distinct risk profiles.

**Table 3:** Meta-regression results of all included studies.

Factors	Variables	Coefficient	Std. err.	*z*	*P*
Unhealthy lifestyle	Unhealthy Habits	0.157	0.061	2.59	0.010
	Pathological Types	−0.017	0.037	−0.45	0.655
	Study Region	0.057	0.022	2.56	0.011
	Age	−0.006	0.009	−0.72	0.469
	Study Year	0.016	0.008	1.91	0.057
Metabolic disease	Diseases	−0.251	0.077	−3.27	0.001
	Pathological Types	−0.028	0.060	−0.47	0.636
	Study Region	0.050	0.107	0.47	0.637
	Age	0.007	0.008	0.91	0.362
	Study Year	−0.002	0.011	−0.22	0.830

### 3.3 Stratified lifestyle factors and smoking-specific analysis

**Figure 2:**
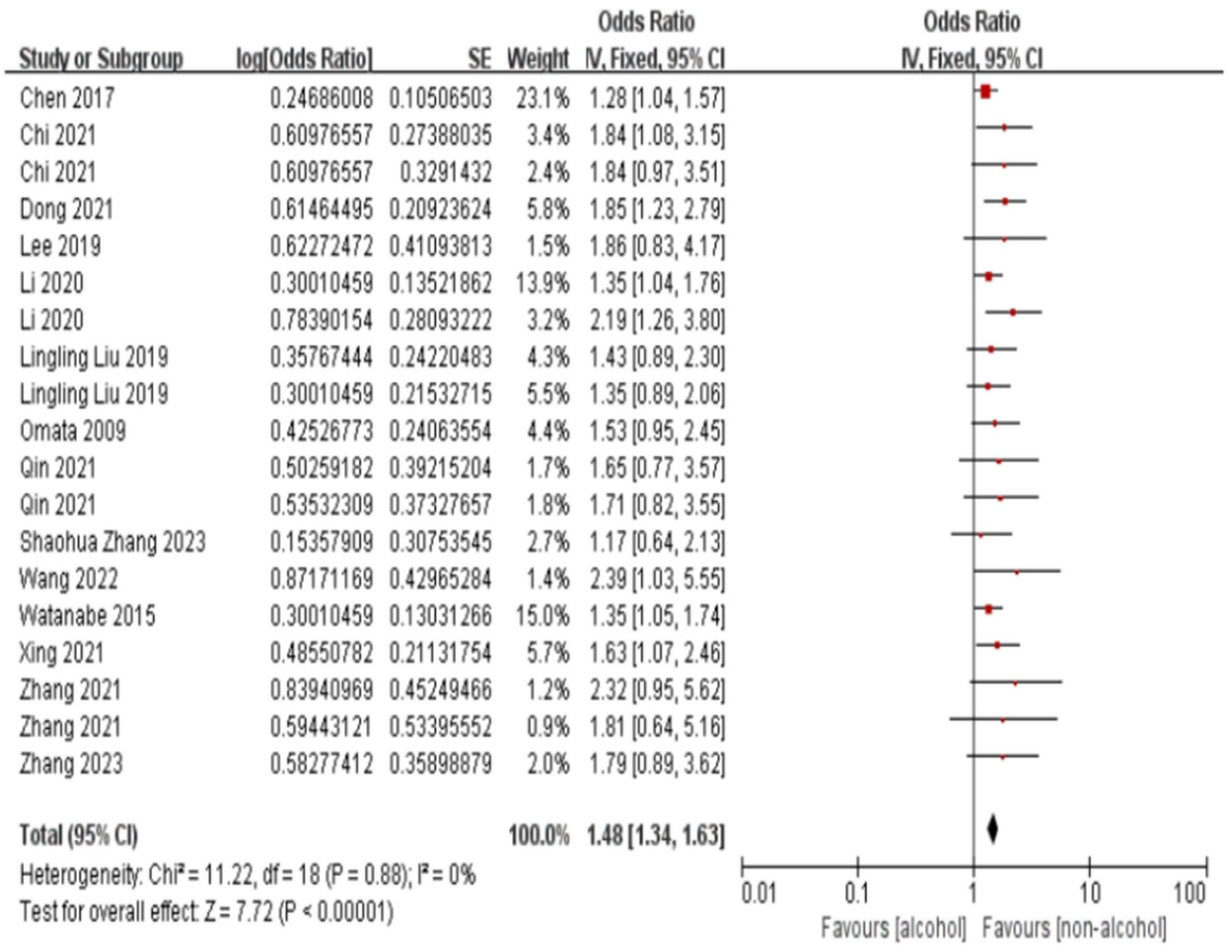
Effect and forest plot of alcohol consumption on colorectal polyps. The forest plot and meta-analysis results on the impact of alcohol consumption on colorectal polyps are shown in the figure.

**Figure 3:**
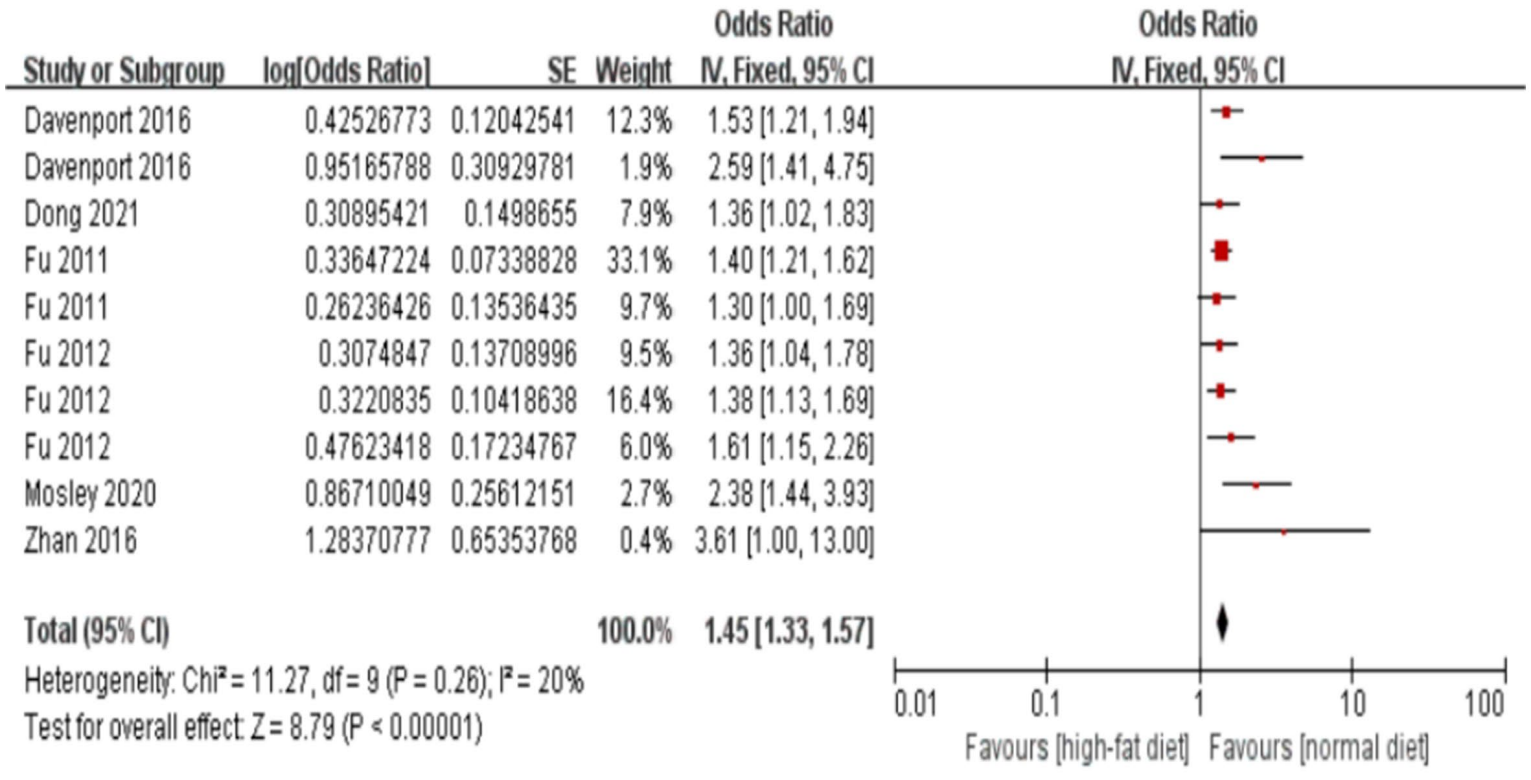
Effect and forest plot of high-fat diet on colorectal polyps. Forest plot and meta-analysis results suggesting that a high-fat diet is a risk factor for colorectal polyps.

The forest plot and meta-analysis results on the impact of alcohol consumption on colorectal polyps are shown in Figure 2. Alcohol consumption demonstrated a consistent risk elevation across 19 studies (OR = 1.63, 95%CI: 1.48–1.78; I^2^ = 0%), with symmetrical funnel plot distribution (Supplementary Figure S1) and robust sensitivity estimates (OR range:1.48–1.56 upon study exclusion). High-fat diet showed consistently moderate association (OR = 1.45, 1.33–1.57; I^2^ = 20%), while maintaining stability in leave-one-out analyses (Supplementary Table S2). In the meanwhile, funnel plot also illustrates no substantial publication bias in the study results (Supplementary Figure S3; Figure 3).

Smoking emerged as the most significant lifestyle risk factor (OR = 1.79, 1.69–1.90; I^2^ = 45%), prompting deeper investigation through meta-regression. Meta-regression in Table 4 demonstrated that pathological types and geographic region were primary heterogeneity sources. Regarding pathological types, with adenoma as the reference category, the meta-regression revealed a significantly stronger association between smoking and serrated sessile lesions (SSLs; coefficient = 0.394, *P* = 0.006). Similarly, the association with “other” pathological types was also significantly stronger compared to adenoma (coefficient = 0.177, *P* = 0.016). In contrast, the association with hyperplastic polyps (HPs) did not significantly differ from that with adenoma (coefficient = 0.356, *P* = 0.232). Neither age (coefficient = 0.004, *P* = 0.387) nor study year (coefficient = 0.005, *P* = 0.387) were found to be significant moderators of the association between smoking and colorectal polyps.

**Table 4:** Meta-regression results of smoking factor studies.

Factors	Variables	Coefficient	Std. err.	*z*	*P*
Pathological types	Adenoma	Ref.			
	SSLs	0.394	0.143	2.763	0.006
	HPs	0.356	0.298	1.204	0.232
	Others	0.177	0.074	2.402	0.016
Age		0.004	0.008	0.471	0.387
Study year		0.005	0.006	0.873	0.387
Study region	China	Ref.			
	the U. S.	0.715	0.121	5.876	<0.001
	Korea	0.569	0.226	2.523	0.012
	Japan	0.006	0.159	0.040	0.970
	Israel	0.750	0.159	4.722	<0.001
	Germany	0.180	0.249	0.721	0.470
	Italy	0.037	0.353	0.110	0.916

**Figure 4:**
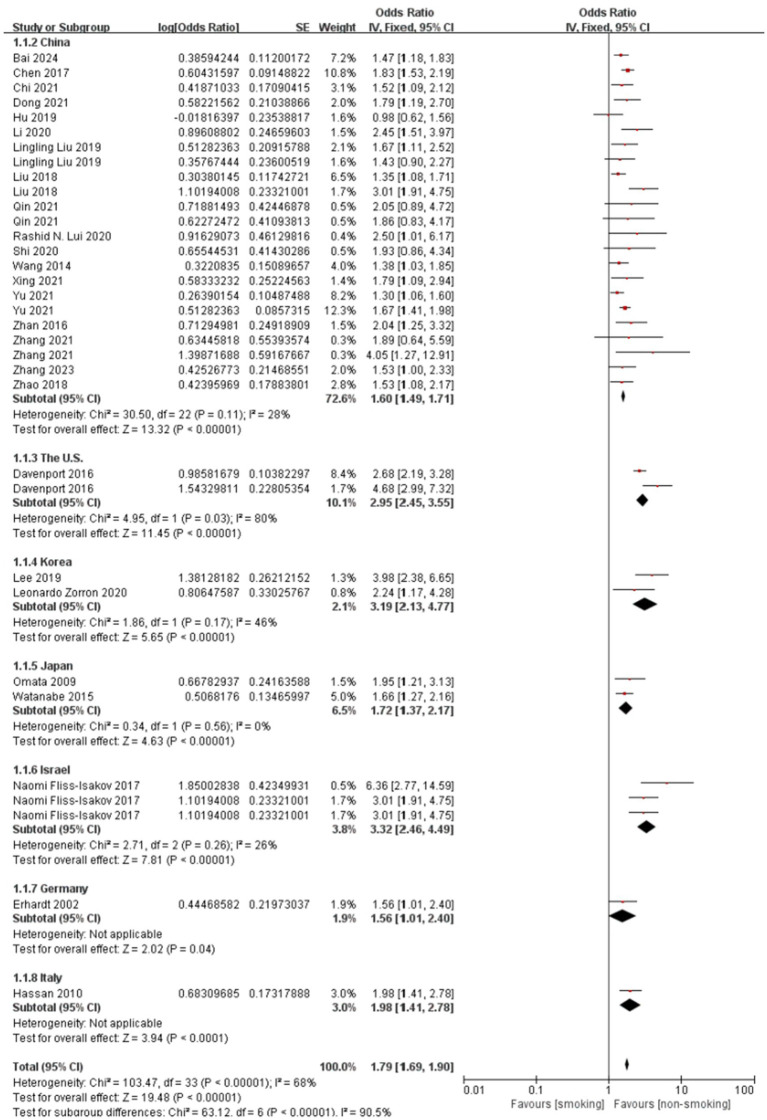
Effect and forest plot of smoking factor on colorectal polyps in subgroups by study region. Meta-analysis result suggesting that smoking markedly increases the risk of colorectal polyps across different regions.

Figure 4 illustrates the subgroup analysis by study region, further elucidating the regional variations in the association between smoking and colorectal polyps. Consistent with the meta-regression findings, studies from the US, Korea, and Israel demonstrated notably higher pooled ORs compared to China, indicating a stronger association in these regions. The test for subgroup differences was highly significant (*P* < 0.001), confirming that study region significantly contributed to the observed overall heterogeneity. Within most regional subgroups, heterogeneity was low or absent (I^2^ ≤ 28%), with the exception of China, which still showed moderate heterogeneity.

**Figure 5:**
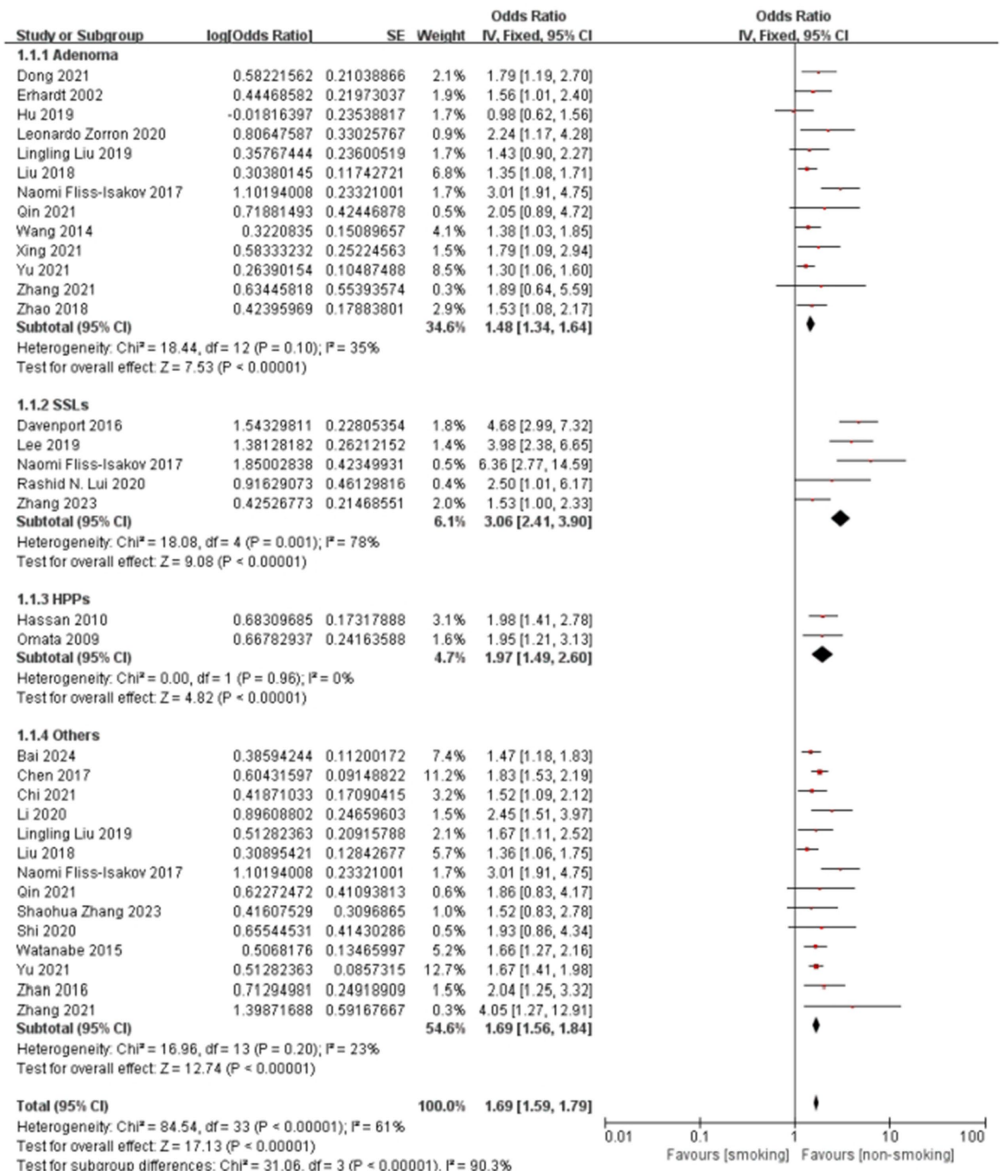
Effect and forest plot of smoking factor on colorectal polyps in subgroups by pathological type. The result smoking significantly increases the risk for all types of colorectal polyps.

Smoking demonstrated pathological subtype specificity in Figure 5. The overall pooled OR for this specific subgroup analysis was 1.69 (95% CI: 1.59–1.79), with substantial heterogeneity (I^2^ = 61%, *P* < 0.001). Subgroup analysis results align with the meta-regression results, showing that the association between smoking and SSLs (pooled OR = 3.06) was considerably stronger than that with adenomas (pooled OR = 1.48). The association with “Others” (pooled OR = 1.69) was also higher than adenomas, though the difference was less pronounced than for SSLs. While HPs showed a higher pooled OR than adenomas, the meta-regression did not find a statistically significant difference, possibly influenced by the limited number of studies (n = 2) for HPs in the subgroup analysis. Significant heterogeneity was observed within the SSLs subgroup (I^2^ = 78%), whereas heterogeneity was low or absent in HPs and “Others” subgroups. The test for subgroup differences was highly significant (*P* < 0.001), confirming pathological type as a significant source of heterogeneity.

The generally symmetrical distribution of points in the funnel plots (Supplementary Figure 6) indicated no significant publication bias, supporting the consistency of the findings. Sensitivity analyses (Supplementary Table 3; Supplementary Figure 7) revealed high consistency for the hyperplastic polyp subgroup, and stable results with low heterogeneity for the adenoma and “other” polyp types subgroups. The serrated lesion subgroup, however, shows higher heterogeneity, suggesting potential differences in study design or population characteristics.

### 3.4 Impact of metabolic diseases on colorectal polyps

**Figure 6:**
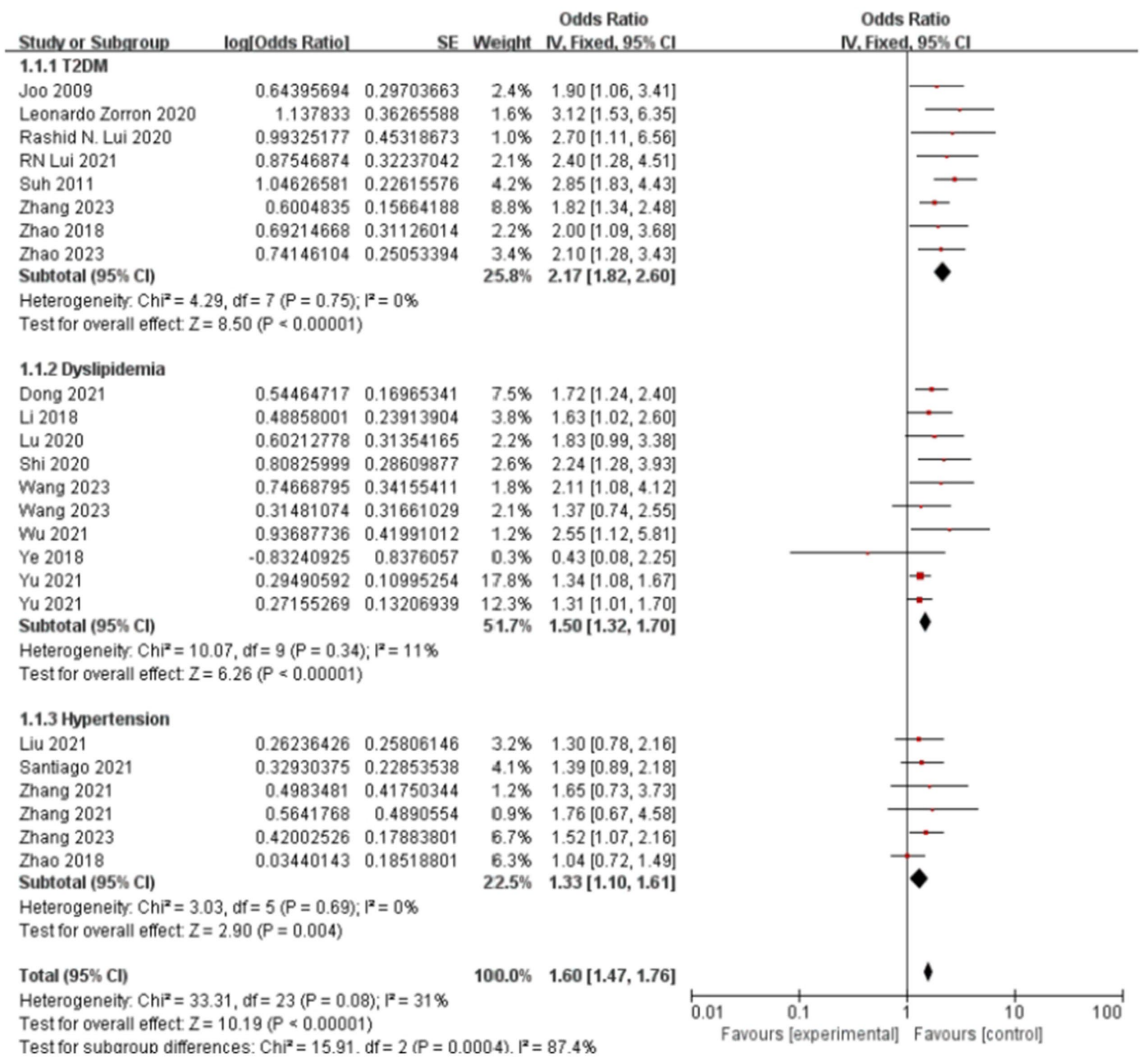
Effect and forest plot of metabolic disease factors on colorectal polyps in each disease subgroup. The meta-analysis results indicated that various metabolic diseases significantly increase the risk of colorectal polyps, with type 2 diabetes mellitus (T2DM) demonstrating the most substantial risk increase.

Figure 6 presents the forest plot of metabolic disease subtypes in relation to colorectal polyp risk. Pooled analysis demonstrated a hierarchical risk pattern: type 2 diabetes mellitus (T2DM) exhibited the strongest association (OR = 2.17, 95%CI: 1.82–2.60), followed by hyperlipidemia (OR = 1.50, 95%CI: 1.32–1.70) and hypertension (OR = 1.33, 95%CI: 1.10–1.61). The T2DM and hypertension subgroups showed complete homogeneity across studies (I^2^ = 0%), while hyperlipidemia analyses displayed moderate heterogeneity (I^2^ = 35%), potentially reflecting variations in diagnostic thresholds between studies.

Methodological validation confirmed result robustness. Funnel plot symmetry (Supplementary Figure 8) indicated minimal publication bias. Sensitivity analyses demonstrated stable effect estimates for T2DM (OR range:2.09–2.41 upon study exclusion) and hypertension (OR range:1.27–1.47), as detailed in Supplementary Table 4. The observed heterogeneity in hyperlipidemia studies (Supplementary Figure 9) may stem from differential adjustments for lipid-lowering medication use across cohorts.

## 4 Discussion

This study, through a meta-analysis, examines the impact of unhealthy lifestyle choices and metabolic diseases on different pathological types of colorectal polyps. Among the studies included, smoking is identified as a significant risk factor for colorectal polyps, representing a key unhealthy lifestyle habit. In subgroup analyses by pathological type, the risk associated with smoking is significantly higher for sessile serrated lesions (SSLs) compared to adenomas. Consistent with this finding, an RCT conducted in the United States found that smoking increased the risk for SSLs more than for microvesicular hyperplastic polyps (MVHP) (13). In agreement with our study, a 2017 meta-analysis also reported a strong association between smoking and SSLs (14). Moreover, a more detailed study suggested that smoking particularly elevates the risk of serrated polyps in the left colon (15). Mechanistic research suggests that smoking may increase the risk of serrated colorectal cancer and polyps by promoting carcinogenic pathways related to microsatellite instability (MSI), CpG island methylator phenotype (CIMP), and/or BRAF mutations (16, 17). Additionally, smoking may impair immune surveillance by affecting immune regulation processes, such as weakening T-cell memory, thus reducing the efficiency of immune monitoring (18–20).

Our findings also indicate that alcohol consumption is another risk factor for colorectal polyps. Once metabolized in the body, alcohol can trigger various molecular responses that lead to colorectal lesions (21, 22). The oxidative and non-oxidative metabolism of alcohol, along with the formation of reactive oxygen species (ROS) and other byproducts, may result in alterations in genetic, epigenetic, cell signaling, and immune processes (23).

A high-fat diet has also been strongly associated with an increased risk of colorectal polyps and even colorectal cancer, likely through mechanisms that promote disease by facilitating bile acid metabolism and gut microbiota interactions (24). Similarly, studies in mice have shown significantly higher polyp incidence in mice on high-fat (HFD) and high-sugar diets (HSD) compared to a normal diet (25). Among metabolic diseases, abnormal lipid metabolism is similarly linked to an increased risk of colorectal polyps. Several studies suggest an elevated risk of colorectal adenoma in patients with metabolic syndrome (8, 26), with a higher risk associated with a greater number of metabolic syndrome components. Insulin resistance, a primary mechanism in metabolic syndrome, also plays a key role in the development of colorectal cancer and polyps (27). Our study supports these findings, showing that type 2 diabetes mellitus (T2DM) is a consistent risk factor for colorectal polyps.

In this study, the combined effect size and 95% confidence interval (CI) for hypertension’s association with colorectal polyps was 1.33 (1.10, 1.61). Numerous epidemiological studies have established a link between metabolic risk factors and colorectal cancer risk (28, 29). In a large-scale study in Australia, hypertension was found to impact different pathological types of colorectal polyps variably, with odds ratios (OR) and 95% CI values for adenomas and sessile serrated polyps (SSPs) at 0.92 (0.48, 1.76) and 0.76 (0.40, 1.43), respectively.

This study has several limitations warranting consideration. Firstly, the analysis relies exclusively on case–control studies, making it susceptible to inherent selection and recall biases; consequently, the reported ORs may overestimate true risk associations. Significant heterogeneity across study populations also tempers the generalizability of our findings. More importantly, the inconsistent diagnostic criteria for key exposures—such as the varying clinical thresholds for hyperlipidemia and hypertension—introduce a substantial risk of non-differential misclassification bias. This systematic error could distort the pooled effect estimates, leading to an underestimation of the strength of the association between exposure and outcome. Furthermore, our subgroup analysis was stratified by geographic region rather than ethnicity. We acknowledge that ethnicity is a more biologically relevant factor than geographic location for assessing risk, particularly for metabolic diseases. However, this more granular analysis was constrained by the lack of ethnicity-specific data in the vast majority of primary studies. Secondly, our strategy of prioritizing peer-reviewed publications over most unpublished gray literature, while intended to ensure data quality, creates a potential for publication bias. However, this concern is substantially mitigated as our funnel plot analyses revealed no significant asymmetry for the primary outcomes.

In summary, unhealthy lifestyle habits (such as alcohol consumption, high-fat diets, and smoking) and metabolic diseases (such as T2DM, hyperlipidemia, and hypertension) significantly increase the risk of colorectal polyps across different pathological types. Smoking, in particular, not only markedly raises the overall incidence of colorectal polyps but also has variable effects depending on pathological type and region. A ‘one-size-fits-all’ global screening guideline may be suboptimal. For instance, public health campaigns and clinical risk assessments in Western and other high-risk nations might warrant placing a greater emphasis on smoking cessation as a primary prevention strategy for colorectal polyps, especially for serrated pathway lesions. Therefore, the prevention and management of these lifestyle habits and metabolic diseases are critical in reducing the risk of colorectal polyps. Further high-quality research is needed to elucidate the specific relationships and underlying mechanisms between these factors and colorectal polyps.

## Footnotes

^1^ http://apps.who.int/trialsearch/
