# Supplementary material for: Modifiable lifestyle and metabolic risk factors for colorectal polyps: a systematic review and meta-analysis
Source: Front Public Health. 2025 Oct 8;13:1655750. doi: 10.3389/fpubh.2025.1655750 (PMC12542907; doi:10.3389/fpubh.2025.1655750)
Supplement: Supplementary file 1 [file Supplementary_file_1.docx]

| **Supplementary Table S1. Sensitivity Analysis Results for the Impact of Alcohol Consumption on Colorectal Polyps** | | | | | | | |
| --- | --- | --- | --- | --- | --- | --- | --- |
| **Excluded Studies** | **logOR** | **SE** | **Statistic** | **OR(95%CI)** | **P** | **tau2** | **I2(%)** |
| Chen 2017 | 0.432 | 0.058 | 7.511 | 1.56 (1.45, 1.68) | <0.001 | <0.001 | <0.001 |
| Chi2021 | 0.382 | 0.051 | 7.433 | 1.49 (1.39, 1.59) | <0.001 | <0.001 | <0.001 |
| Chi2021 | 0.384 | 0.051 | 7.521 | 1.49 (1.39, 1.59) | <0.001 | <0.001 | <0.001 |
| Dong 2021 | 0.376 | 0.052 | 7.220 | 1.48 (1.37, 1.58) | <0.001 | <0.001 | <0.001 |
| Lee 2019 | 0.386 | 0.051 | 7.587 | 1.49 (1.39, 1.59) | <0.001 | <0.001 | <0.001 |
| Li 2020 | 0.412 | 0.057 | 7.270 | 1.52 (1.42, 1.63) | <0.001 | <0.001 | <0.001 |
| Li 2020 | 0.376 | 0.051 | 7.334 | 1.48 (1.38, 1.58) | <0.001 | <0.001 | <0.001 |
| Lingling Liu 2019 | 0.391 | 0.052 | 7.574 | 1.50 (1.40, 1.60) | <0.001 | <0.001 | <0.001 |
| Lingling Liu 2019 | 0.396 | 0.052 | 7.582 | 1.51 (1.41, 1.61) | <0.001 | <0.001 | <0.001 |
| Omata 2009 | 0.388 | 0.052 | 7.512 | 1.50 (1.40, 1.60) | <0.001 | <0.001 | <0.001 |
| Qin 2021 | 0.388 | 0.051 | 7.614 | 1.50 (1.40, 1.60) | <0.001 | <0.001 | <0.001 |
| Qin 2021 | 0.387 | 0.051 | 7.592 | 1.49 (1.40, 1.59) | <0.001 | <0.001 | <0.001 |
| Shaohua Zhang 2023 | 0.396 | 0.051 | 7.739 | 1.51 (1.41, 1.61) | <0.001 | <0.001 | <0.001 |
| Wang 2022 | 0.383 | 0.051 | 7.529 | 1.49 (1.39, 1.59) | <0.001 | <0.001 | <0.001 |
| Watanabe 2015 | 0.413 | 0.057 | 7.247 | 1.53 (1.42, 1.63) | <0.001 | <0.001 | <0.001 |
| Xing 2021 | 0.384 | 0.052 | 7.381 | 1.49 (1.39, 1.59) | <0.001 | <0.001 | <0.001 |
| Zhang 2021 | 0.384 | 0.051 | 7.556 | 1.49 (1.39, 1.59) | <0.001 | <0.001 | <0.001 |
| Zhang 2021 | 0.388 | 0.051 | 7.645 | 1.49 (1.39, 1.59) | <0.001 | <0.001 | <0.001 |
| Zhang 2023 | 0.386 | 0.051 | 7.563 | 1.50 (1.39, 1.60) | <0.001 | <0.001 | <0.001 |
| Pooled estimate | 0.390 | 0.050 | 7.716 | 1.50 (1.40, 1.60) | <0.001 | <0.001 | <0.001 |

| 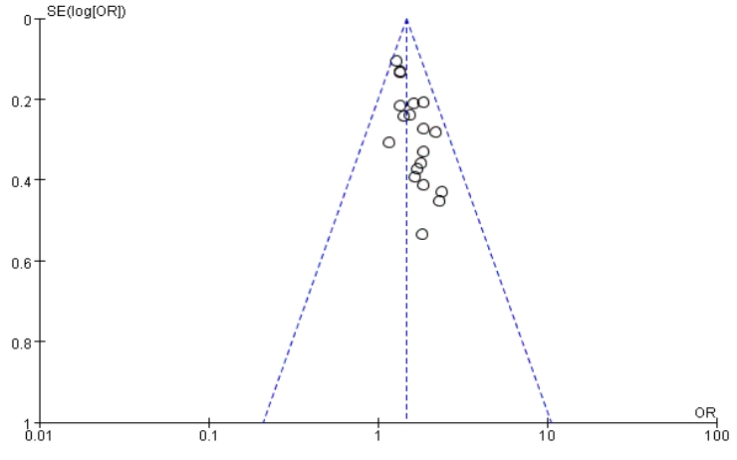 |
| --- |
| **Supplementary Figure S1**. Funnel Plot of Alcohol Consumption Factor |
| ***Note****: The funnel plot for the impact of alcohol consumption on colorectal polyps suggesting no substantial publication bias in the study results.* |

| 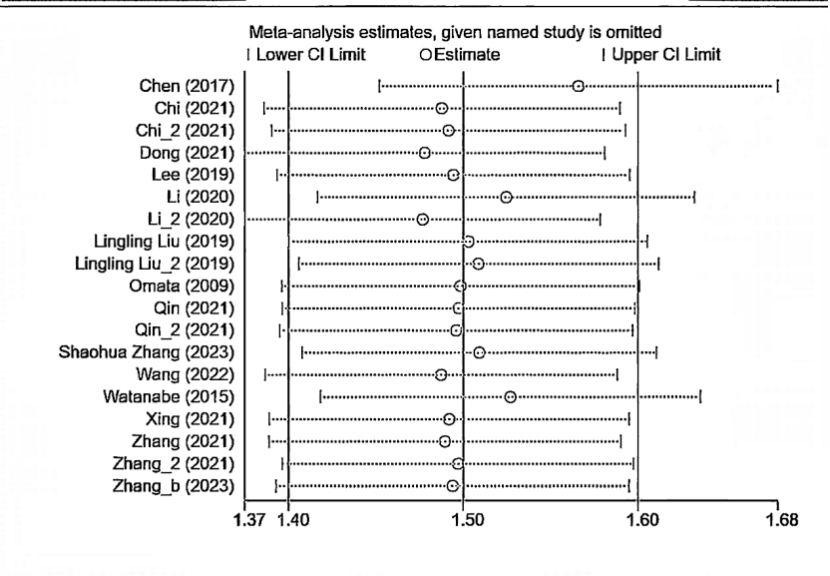 |
| --- |
| **Supplementary Figure S2.** Sensitivity Analysis Plot for the Impact of Alcohol Consumption on Colorectal Polyps |
| ***Note:*** *Sensitivity analysis supported the robustness of the results.* |

| **Supplementary Table S2**. Sensitivity Analysis Results for Meta Analysis on the association of High-Fat Diet Impact on Colorectal Polyps | | | | | | | |
| --- | --- | --- | --- | --- | --- | --- | --- |
| **Excluded Studies** | **logOR** | **SE** | **Statistic** | **OR(95%CI)** | ***P*** | **tau^2^** | ***I^2^* (%)** |
| Davenport 2016 | 0.364 | 0.045 | 8.061 | 1.46 (1.37, 1.55) | <0.001 | <0.001 | 27.562 |
| Davenport 2016 | 0.360 | 0.043 | 8.446 | 1.45 (1.36, 1.53) | <0.001 | <0.001 | <0.001 |
| Dong 2021 | 0.376 | 0.044 | 8.552 | 1.48 (1.39, 1.60) | <0.001 | <0.001 | 27.847 |
| Fu 2011 | 0.388 | 0.052 | 7.519 | 1.50 (1.40, 1.60) | <0.001 | <0.001 | 26.883 |
| Fu 2011 | 0.383 | 0.044 | 8.611 | 1.48 (1.40, 1.57) | <0.001 | <0.001 | 24.238 |
| Fu 2012 | 0.378 | 0.044 | 8.509 | 1.48 (1.39, 1.56) | <0.001 | <0.001 | 27.513 |
| Fu 2012 | 0.381 | 0.046 | 8.241 | 1.48 (1.39, 1.57) | <0.001 | <0.001 | 27.335 |
| Fu 2012 | 0.364 | 0.044 | 8.365 | 1.46 (1.37, 1.54) | <0.001 | <0.001 | 26.462 |
| Mosley 2020 | 0.357 | 0.043 | 8.344 | 1.44 (1.36, 1.52) | <0.001 | <0.001 | <0.001 |
| Zhan 2016 | 0.367 | 0.042 | 8.679 | 1.46 (1.37, 1.54) | <0.001 | <0.001 | 14.130 |
| Pooled estimate | 0.371 | 0.042 | 8.787 | 1.47 (1.38, 1.55) | <0.001 | <0.001 | 20.174 |

| 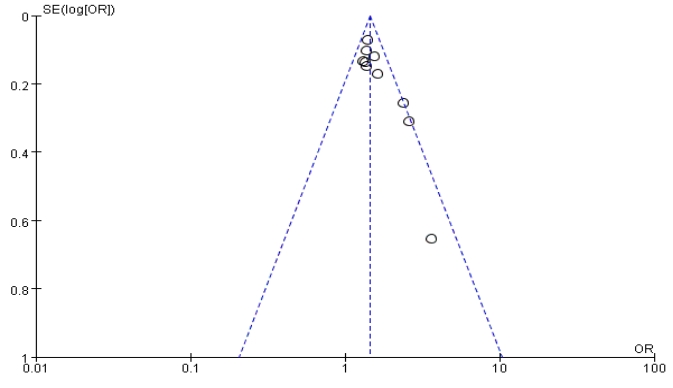 |
| --- |
| **Supplementary Figure S3.** Funnel Plot of High-Fat Diet Factor |
| ***Note:*** *The funnel plot for the impact of High-fat Diet on colorectal polyps suggesting no substantial publication bias in the study results.* |

| 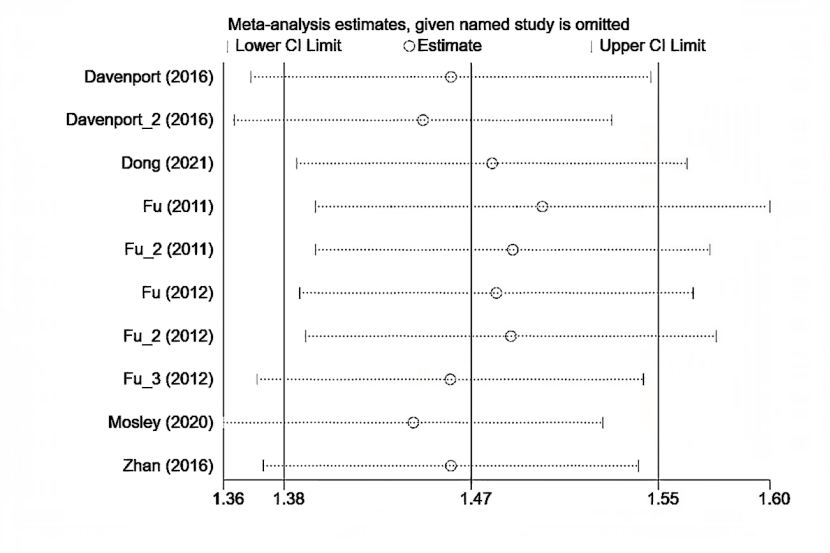 |
| --- |
| **Supplementary Figure S4**. Sensitivity Analysis Plot for the Impact of High-Fat Diet on Colorectal Polyps |
| ***Note:*** *The result of sensitivity analysis demonstrating low to moderate heterogeneity, indicating that excluding individual studies had a limited impact on the overall effect size.* |

| 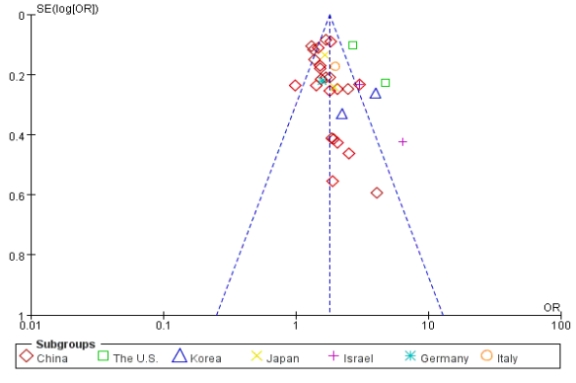 |
| --- |
| **Supplementary Figure S5.** Funnel Plot of Smoking Factor (Study Region Subgroup) |
| ***Note:*** *The symmetry and uniform distribution of points in the funnel plot, indicating a high level of credibility.* |

| 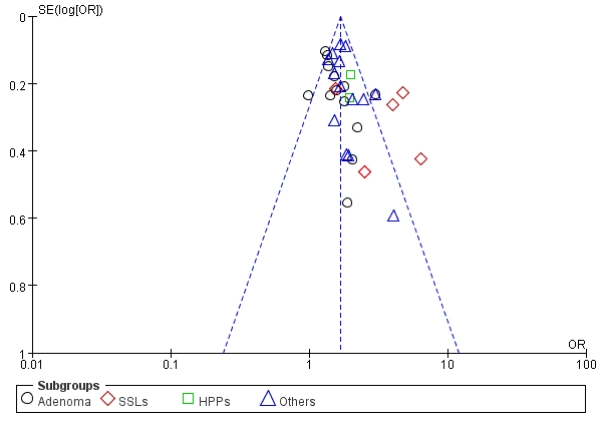 |
| --- |
| **Supplementary Figure S6**. Funnel Plot of Smoking Factor (Pathological Type Subgroup) |
| ***Note:*** *The symmetrical and uniform distribution of points indicates no significant publication bias in the study results.* |

| **Supplementary Table S3.** Sensitivity Analysis Results for the Impact of Smoking on Colorectal Polyps in Pathological Type Subgroups | | | | | | | | |
| --- | --- | --- | --- | --- | --- | --- | --- | --- |
| **Pathological Types** | **Excluded Studies** | **logOR** | **SE** | **Statistic** | **OR(95%CI)** | **P** | **tau^2^** | **I^2^(%)** |
| Adenoma | Dong 2021 | 0.421 | 0.076 | 5.510 | 1.50 (1.40, 1.61) | <0.01 | 0.024 | 37.355 |
|  | Erhardt 2002 | 0.437 | 0.079 | 5.511 | 1.52 (1.41, 1.62) | <0.01 | 0.029 | 40.133 |
|  | Hu 2019 | 0.458 | 0.071 | 6.410 | 1.55 (1.44, 1.65) | <0.01 | 0.018 | 27.900 |
|  | Leonardo Zorron 2020 | 0.413 | 0.071 | 5.829 | 1.50 (1.40, 1.60) | <0.01 | 0.019 | 34.586 |
|  | Lingling Liu 2019 | 0.443 | 0.079 | 5.618 | 1.52 (1.42, 1.63) | <0.01 | 0.029 | 40.268 |
|  | Liu 2018 | 0.459 | 0.083 | 5.532 | 1.56 (1.45, 1.67) | <0.01 | 0.031 | 38.018 |
|  | Naomi Fliss-Isakov 2015 | 0.354 | 0.053 | 6.640 | 1.44 (1.34, 1.55) | <0.01 | <0.01 | <0.01 |
|  | Qin 2021 | 0.424 | 0.073 | 5.836 | 1.51 (1.41, 1.61) | <0.01 | 0.022 | 38.314 |
|  | Wang 2014 | 0.454 | 0.082 | 5.508 | 1.54 (1.43, 1.65) | <0.01 | 0.031 | 39.555 |
|  | Xing 2021 | 0.424 | 0.076 | 5.601 | 1.51 (1.40, 1.61) | <0.01 | 0.024 | 38.310 |
|  | Yu 2021 | 0.465 | 0.080 | 5.776 | 1.59 (1.47, 1.71) | <0.01 | 0.026 | 33.261 |
|  | Zhang 2021 | 0.428 | 0.073 | 5.899 | 1.52 (1.41, 1.62) | <0.01 | 0.023 | 39.701 |
|  | Zhao 2018 | 0.440 | 0.082 | 5.402 | 1.52 (1.41, 1.62) | <0.01 | 0.031 | 40.218 |
|  | Pooled estimate | 0.431 | 0.072 | 6.025 | 1.52 (1.42, 1.62) | <0.01 | 0.022 | 34.913 |
| SSLs | Davenport 2016 | 1.101 | 0.318 | 3.462 | 2.97 (2.69, 3.26) | 0.001 | 0.290 | 77.276 |
|  | Lee 2019 | 1.156 | 0.334 | 3.459 | 3.33 (3.05, 3.60) | 0.001 | 0.336 | 82.141 |
|  | Naomi Fliss-Isakov 2015 | 1.073 | 0.280 | 3.837 | 3.20 (2.95, 3.46) | <0.01 | 0.230 | 79.768 |
|  | Rashid N. Lui 2020 | 1.257 | 0.312 | 4.023 | 3.55 (3.30, 3.80) | <0.01 | 0.310 | 83.210 |
|  | Zhang 2023 | 1.462 | 0.151 | 9.702 | 4.43 (4.13, 4.72) | <0.01 | <0.01 | 0.000 |
|  | Pooled estimate | 1.199 | 0.262 | 4.578 | 3.47 (3.23, 3.71) | <0.01 | 0.244 | 77.873 |
| HPs | Omata 2009 | 0.683 | 0.173 | 3.944 | 1.95 (1.61, 2.29) | <0.01 | - | - |
|  | Hassan 2010 | 0.668 | 0.242 | 2.764 | 1.98 (1.51, 2.45) | 0.006 | - | - |
|  | Pooled estimate | 0.678 | 0.141 | 4.816 | 1.96 (1.68, 2.24) | <0.01 | <0.01 | <0.01 |

| 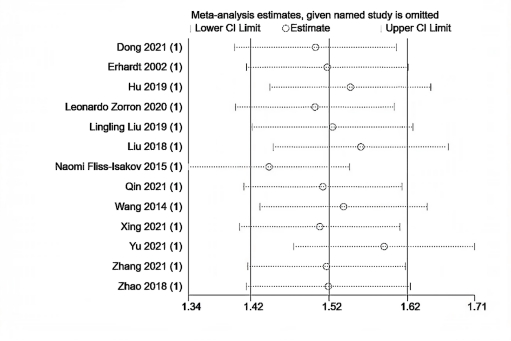 | 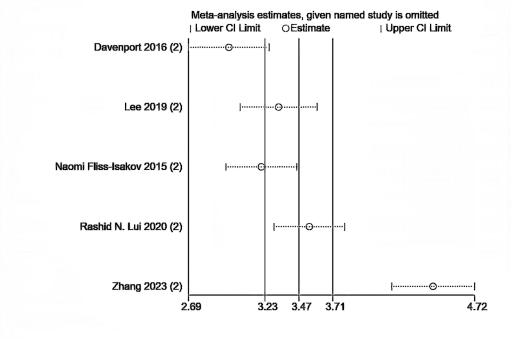 |
| --- | --- |
| 1. Sensitivity Analysis Plot for Adenoma Subgroup | 1. Sensitivity Analysis Plot for SSPs Subgroup |
| 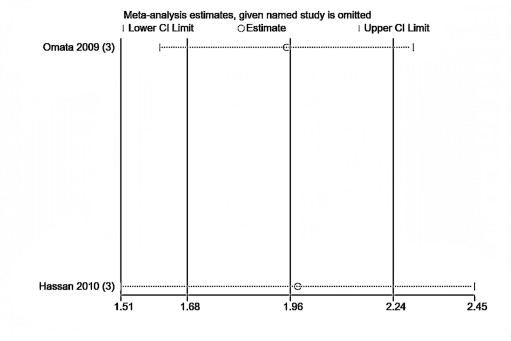 | 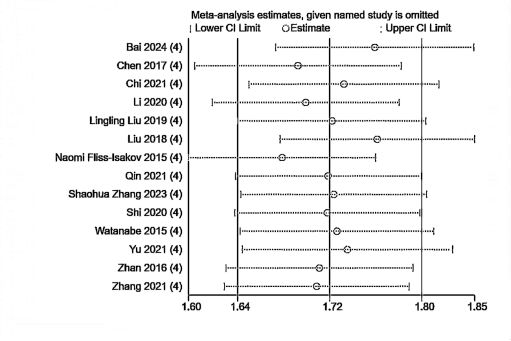 |
| 1. Sensitivity Analysis Plot for HPs Subgroup | 1. Sensitivity Analysis Plot for Others Subgroup |
| **Supplementary Figure S7.** Sensitivity Analysis Plot for the Impact of Smoking on Colorectal Polyps in Pathological Type Subgroups | |

| 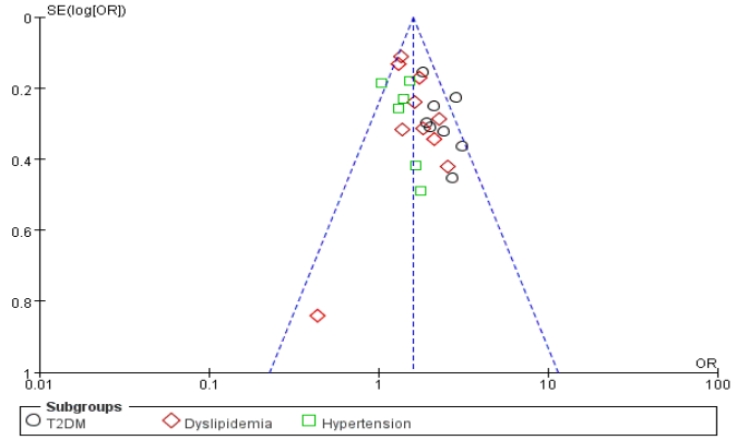 |
| --- |
| **Supplementary Figure S8.** Funnel Plot of Metabolic Disease Factors |
| *Note: Funnel Plot supported the reliability and comprehensiveness of the included studies.* |

| 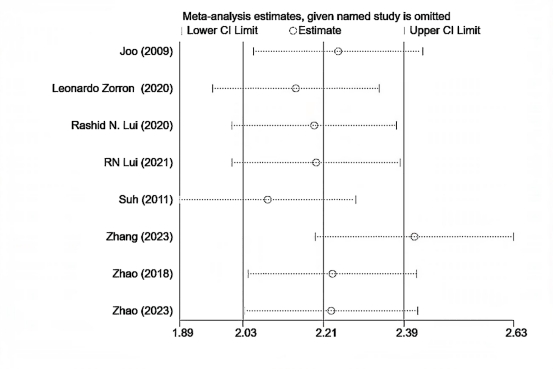 |
| --- |
| 1. Sensitivity Analysis Plot for T2DM Subgroup |
| 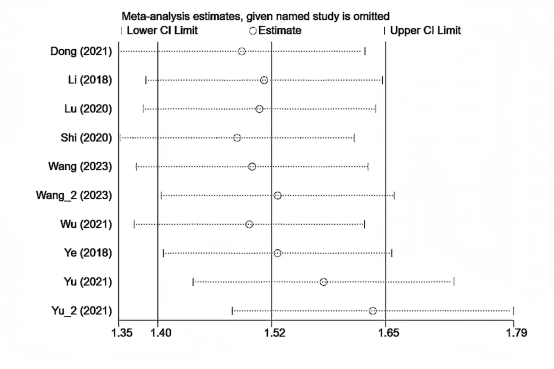 |
| 1. Sensitivity Analysis Plot for Dyslipidemia Subgroup |
| 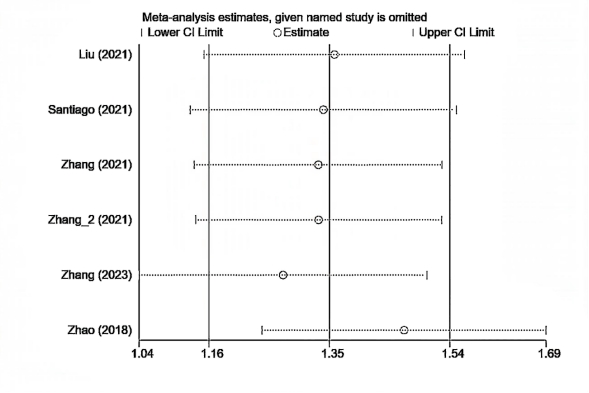 |
| 1. Sensitivity Analysis Plot for Hypertension Subgroup |
| **Supplementary Figure 9.** Sensitivity Analysis Plot of the Impact of Metabolic Diseases on Colorectal Polyps in Disease Subgroups |

| **Supplementary Table 4.** Sensitivity Analysis Results for Meta Analysis of Metabolic Diseases Impact on Colorectal Polyps in Disease Subgroups | | | | | | | | |
| --- | --- | --- | --- | --- | --- | --- | --- | --- |
| Metabolic Diseases | Excluded Studies | logOR | SE | Statistic | OR(95%CI) | *P* | tau^2^ | *I^2^* (%) |
| T2DM | Zhao2018 | 0.788 | 0.099 | 8.002 | 2.25 (2.06, 2.43) | <0.001 | 0.003 | <0.001 |
|  | RashidN.Lui2021 | 0.767 | 0.093 | 8.226 | 2.15 (1.97, 2.34) | <0.001 | 0.000 | <0.001 |
|  | RNLui2021 | 0.768 | 0.096 | 8.029 | 2.19 (2.01, 2.37) | <0.001 | <0.001 | <0.001 |
|  | Zhang2023 | 0.866 | 0.112 | 7.709 | 2.20 (2.01, 2.38) | <0.001 | <0.001 | <0.001 |
|  | Zhao2023 | 0.789 | 0.103 | 7.647 | 2.09 (1.89, 2.28) | <0.001 | 0.005 | <0.001 |
|  | Joo2009 | 0.795 | 0.100 | 7.992 | 2.41 (2.19, 2.63) | <0.001 | 0.003 | <0.001 |
|  | Suh2011 | 0.723 | 0.100 | 7.248 | 2.32 (2.05, 2.42) | <0.001 | <0.001 | <0.001 |
|  | LeonardoZorron2020 | 0.751 | 0.094 | 7.966 | 2.23 (2.04, 2.42) | <0.001 | <0.001 | <0.001 |
|  | Pooled estimate | 0.776 | 0.091 | 8.499 | 2.21 (2.03, 2.39) | <0.001 | <0.001 | <0.001 |
| Dyslipidemia | Dong2021 | 0.380 | 0.070 | 5.452 | 1.49 (1.35, 1.63) | <0.001 | <0.001 | 13.708 |
|  | Yu2021 | 0.473 | 0.087 | 5.459 | 1.52 (1.38, 1.65) | <0.001 | 0.009 | 8.594 |
|  | Li2018 | 0.417 | 0.077 | 5.412 | 1.51 (1.38, 1.64) | <0.001 | 0.007 | 19.509 |
|  | Lu2020 | 0.406 | 0.071 | 5.690 | 1.49 (1.36, 1.62) | <0.001 | 0.003 | 17.159 |
|  | Shi2020 | 0.382 | 0.066 | 5.775 | 1.50 (1.37, 1.63) | <0.001 | <0.001 | <0.001 |
|  | Wang2023 | 0.395 | 0.067 | 5.854 | 1.53 (1.40, 1.66) | <0.001 | 0.001 | 11.408 |
|  | Wang2023 | 0.431 | 0.077 | 5.611 | 1.50 (1.37, 1.63) | <0.001 | 0.008 | 19.928 |
|  | Wu2021 | 0.392 | 0.066 | 5.958 | 1.53 (1.40, 1.66) | <0.001 | <0.001 | 5.041 |
|  | Ye2018 | 0.430 | 0.073 | 5.907 | 1.58 (1.44, 1.73) | <0.001 | 0.006 | <0.001 |
|  | Yu2021 | 0.477 | 0.088 | 5.413 | 1.64 (1.48, 1.79) | <0.001 | 0.007 | 6.668 |
|  | Pooled estimate | 0.416 | 0.070 | 5.925 | 1.52 (1.40, 1.65) | <0.001 | 0.004 | 10.660 |
| Hypertension | Zhang2021 | 0.271 | 0.101 | 2.691 | 1.36 (1.15, 1.56) | 0.007 | <0.001 | <0.001 |
|  | Zhao2018 | 0.380 | 0.115 | 3.294 | 1.34 (1.13, 1.55) | 0.001 | <0.001 | <0.001 |
|  | Liu2021 | 0.290 | 0.112 | 2.588 | 1.33 (1.13, 1.53) | 0.010 | 0.005 | <0.001 |
|  | Zhang2023 | 0.225 | 0.117 | 1.924 | 1.33 (1.13, 1.53) | 0.054 | <0.001 | <0.001 |
|  | Zhang2021 | 0.272 | 0.100 | 2.720 | 1.27 (1.04, 1.50) | 0.007 | <0.001 | <0.001 |
|  | Santiago2021 | 0.276 | 0.115 | 2.392 | 1.47 (1.24, 1.69) | 0.017 | 0.006 | <0.001 |
|  | Pooled estimate | 0.283 | 0.098 | 2.895 | 1.35 (1.16, 1.54) | 0.004 | <0.001 | <0.001 |

| **Section and Topic** | **Item #** | **Checklist item** | **Location where item is reported** |
| --- | --- | --- | --- |
| **TITLE** | | |  |
| Title | 1 | Identify the report as a systematic review. | The Impact of Unhealthy Lifestyle Habits and Metabolic Diseases on Colorectal Polyps: A Meta-Analysis and Systematic Review |
| **ABSTRACT** | | |  |
| Abstract | 2 | See the PRISMA 2020 for Abstracts checklist. | Abstract includes structured sections: Objectives, Methods, Results, and Conclusions |
| **INTRODUCTION** | | |  |
| Rationale | 3 | Describe the rationale for the review in the context of existing knowledge. | The review examines the impact of lifestyle and metabolic factors on colorectal polyp risk, highlighting its relevance for prevention. |
| Objectives | 4 | Provide an explicit statement of the objective(s) or question(s) the review addresses. | To assess the relationship between lifestyle habits, metabolic diseases, and colorectal polyps. |
| **METHODS** | | |  |
| Eligibility criteria | 5 | Specify the inclusion and exclusion criteria for the review and how studies were grouped for the syntheses. | Detailed inclusion/exclusion criteria based on population, study design, and outcomes. [1.2 Inclusion and Exclusion Criteria] |
| Information sources | 6 | Specify all databases, registers, websites, organisations, reference lists and other sources searched or consulted to identify studies. Specify the date when each source was last searched or consulted. | Databases searched: PubMed, Embase, Cochrane Library, and Chinese Biomedical Literature Database up to July 2024. [1.1 Search Strategy] |
| Search strategy | 7 | Present the full search strategies for all databases, registers and websites, including any filters and limits used. | Keywords and filters used for database searches are provided. [1.1 Search Strategy] |
| Selection process | 8 | Specify the methods used to decide whether a study met the inclusion criteria of the review, including how many reviewers screened each record and each report retrieved, whether they worked independently, and if applicable, details of automation tools used in the process. | Three researchers independently screened the literature using NoteExpress or Zotero software. [1.3 Data Collection and Quality Assessment] |
| Data collection process | 9 | Specify the methods used to collect data from reports, including how many reviewers collected data from each report, whether they worked independently, any processes for obtaining or confirming data from study investigators, and if applicable, details of automation tools used in the process. | Three researchers independently screened the literature, Any discrepancies were discussed with a third researcher to reach a consensus. [1.3 Data Collection and Quality Assessment] |
| Data items | 10a | List and define all outcomes for which data were sought. Specify whether all results that were compatible with each outcome domain in each study were sought (e.g. for all measures, time points, analyses), and if not, the methods used to decide which results to collect. | [1.1 Search Strategy]  [Table 1] |
|  | 10b | List and define all other variables for which data were sought (e.g. participant and intervention characteristics, funding sources). Describe any assumptions made about any missing or unclear information. | [1.1 Search Strategy]  [Table 1] |
| Study risk of bias assessment | 11 | Specify the methods used to assess risk of bias in the included studies, including details of the tool(s) used, how many reviewers assessed each study and whether they worked independently, and if applicable, details of automation tools used in the process. | Risk of cross-sectional studies assessed using the Newcastle-Ottawa Scale. [1.3 Data Collection and Quality Assessment] |
| Effect measures | 12 | Specify for each outcome the effect measure(s) (e.g. risk ratio, mean difference) used in the synthesis or presentation of results. | Quantitative pooling of effect sizes was performed and expressed as odds ratios (OR) with corresponding 95% confidence intervals (95% CIs).  [1.3 Data Collection and Quality Assessment]  [1.4 Statistical Analysis]  [Table 1] |
| Synthesis methods | 13a | Describe the processes used to decide which studies were eligible for each synthesis (e.g. tabulating the study intervention characteristics and comparing against the planned groups for each synthesis (item #5)). | The study design of all inclueded studies are cross-sectional study.  [Table 1]  [1.4 Statistical Analysis] |
|  | 13b | Describe any methods required to prepare the data for presentation or synthesis, such as handling of missing summary statistics, or data conversions. | [1.4 Statistical Analysis] |
|  | 13c | Describe any methods used to tabulate or visually display results of individual studies and syntheses. | [1.4 Statistical Analysis] |
|  | 13d | Describe any methods used to synthesize results and provide a rationale for the choice(s). If meta-analysis was performed, describe the model(s), method(s) to identify the presence and extent of statistical heterogeneity, and software package(s) used. | [1.4 Statistical Analysis] |
|  | 13e | Describe any methods used to explore possible causes of heterogeneity among study results (e.g. subgroup analysis, meta-regression). | [1.4 Statistical Analysis] |
|  | 13f | Describe any sensitivity analyses conducted to assess robustness of the synthesized results. | [1.4 Statistical Analysis] |
| Reporting bias assessment | 14 | Describe any methods used to assess risk of bias due to missing results in a synthesis (arising from reporting biases). | [1.4 Statistical Analysis] |
| Certainty assessment | 15 | Describe any methods used to assess certainty (or confidence) in the body of evidence for an outcome. | [1.4 Statistical Analysis] |
| **RESULTS** | | |  |
| Study selection | 16a | Describe the results of the search and selection process, from the number of records identified in the search to the number of studies included in the review, ideally using a flow diagram. | Flow diagram showing studies included/excluded with reasons.  [Figure 1] |
|  | 16b | Cite studies that might appear to meet the inclusion criteria, but which were excluded, and explain why they were excluded. |  |
| Study characteristics | 17 | Cite each included study and present its characteristics. | [Table 1] |
| Risk of bias in studies | 18 | Present assessments of risk of bias for each included study. | [Figure 3, Figure 6, Figure 9, Figure 11, Figure 14] |
| Results of individual studies | 19 | For all outcomes, present, for each study: (a) summary statistics for each group (where appropriate) and (b) an effect estimate and its precision (e.g. confidence/credible interval), ideally using structured tables or plots. | [Table 1] |
| Results of syntheses | 20a | For each synthesis, briefly summarise the characteristics and risk of bias among contributing studies. | [Figure 4, Figure 7, Figure 12, Figure 15] |
|  | 20b | Present results of all statistical syntheses conducted. If meta-analysis was done, present for each the summary estimate and its precision (e.g. confidence/credible interval) and measures of statistical heterogeneity. If comparing groups, describe the direction of the effect. | [Figure 2, Figure 5, Figure 8, Figure 10, Figure 13] |
|  | 20c | Present results of all investigations of possible causes of heterogeneity among study results. | [Table 3, Table 6] |
|  | 20d | Present results of all sensitivity analyses conducted to assess the robustness of the synthesized results. | [Table 4, Table 5, Table 7, Table 8] |
| Reporting biases | 21 | Present assessments of risk of bias due to missing results (arising from reporting biases) for each synthesis assessed. | [Table 4, Table 5, Table 7, Table 8] |
| Certainty of evidence | 22 | Present assessments of certainty (or confidence) in the body of evidence for each outcome assessed. | [Table 4, Table 5, Table 7, Table 8] |
| **DISCUSSION** | | |  |
| Discussion | 23a | Provide a general interpretation of the results in the context of other evidence. | [3.Discussion] |
|  | 23b | Discuss any limitations of the evidence included in the review. | [3.Discussion] |
|  | 23c | Discuss any limitations of the review processes used. | [3.Discussion] |
|  | 23d | Discuss implications of the results for practice, policy, and future research. | [3.Discussion] |
| **OTHER INFORMATION** | | |  |
| Registration and protocol | 24a | Provide registration information for the review, including register name and registration number, or state that the review was not registered. | Registered with PROSPERO under ID CRD42024552808 |
|  | 24b | Indicate where the review protocol can be accessed, or state that a protocol was not prepared. |  |
|  | 24c | Describe and explain any amendments to information provided at registration or in the protocol. |  |
| Support | 25 | Describe sources of financial or non-financial support for the review, and the role of the funders or sponsors in the review. | This work was supported by scientific research Foundation of China Human Health Science and Technology Promotion Association(JKHY2023006).  [Funding] |
| Competing interests | 26 | Declare any competing interests of review authors. | There is no competing interests of review authors. |
| Availability of data, code and other materials | 27 | Report which of the following are publicly available and where they can be found: template data collection forms; data extracted from included studies; data used for all analyses; analytic code; any other materials used in the review. | All data are publicly available. |

*From:*  Page MJ, McKenzie JE, Bossuyt PM, Boutron I, Hoffmann TC, Mulrow CD, et al. The PRISMA 2020 statement: an updated guideline for reporting systematic reviews. BMJ 2021;372:n71. doi: 10.1136/bmj.n71. This work is licensed under CC BY 4.0. To view a copy of this license, visit <https://creativecommons.org/licenses/by/4.0/>
